# Mild Cognitive Impairment and Alzheimer’s Disease and Related Disorders

**DOI:** 10.1093/geroni/igaf122.1908

**Published:** 2025-12-31

## Abstract

This session will address both biological and clinical biomarkers of MCI and Alzheimer’s Dementia and Related Dementias

